# A review of the role of *Haemophilus influenzae* in community-acquired pneumonia

**DOI:** 10.15172/pneu.2015.6/520

**Published:** 2015-12-01

**Authors:** Mary P. E. Slack

**Affiliations:** 15 Gold Coast Campus, Griffith University, Queensland, Australia; 25 Institute of Hygiene and Microbiology, University of Würzburg, Würzburg, Germany

**Keywords:** pneumonia, *H. influenzae*, NTHi, aetology, infecton

## Abstract

In an era when *Haemophilus influenzae* type b (Hib) conjugate vaccine is widely used, the incidence of Hib as a cause of community-acquired pneumonia (CAP) has dramatcally declined. Non-typeable *H. influenzae* (NTHi) strains and, occasionally, other encapsulated serotypes of *H. influenzae* are now the cause of the majority of invasive *H. influenzae* infectons, including bacteraemic CAP. NTHi have long been recognised as an important cause of lower respiratory tract infecton, including pneumonia, in adults, especially those with underlying diseases. The role of NTHi as a cause of non-bacteraemic CAP in children is less clear. In this review the evidence for the role of NTHi and capsulated strains of *H. influenzae* will be examined.

## 1. Introduction

Community-acquired pneumonia (CAP) is a major cause of morbidity and mortality in children and adults. Those at the greatest risk are young children in developing countries [1], the elderly, and those with chronic respiratory disease. The implementation of pneumococcal and *Haemophilus influenzae* type b (Hib) conjugate vaccination in many countries has resulted in a reduction in the global number of CAP episodes in young children over the past decade, but pneumonia remains a leading cause of morbidity and mortality in this age group [2]. Walker et al [3] estimated that in 2010 there were 120 million episodes of pneumonia worldwide, 14 million of which progressed to severe pneumonia requiring hospitalisation [4], and 1.3 million deaths in children aged less than 5 years [3], of which 81% occurred in the first two years of life [3]. Pneumonia, particularly severe and recurrent pneumonia, in young children is a risk factor for chronic suppurative lung disease and bronchiectasis in childhood [5–7], which are both debilitating and risk factors for premature death [8]. *Streptococcus pneumoniae* is the most commonly identified bacterial pathogen in CAP in both children and adults, followed by *Mycoplasma pneumoniae, H. influenzae, Chlamydophila pneumoniae* and *Legionella pneumophila* [9]. Both capsulated and non-capsulated or non-typeable strains of *H. influenzae* (NTHi) can cause pneumonia. Hib was one of the most common causes of invasive *H. influenzae* infection, including bacteraemic pneumonia in young children, whereas NTHi was generally considered to be a major cause of chronic respiratory infections and pneumonia in adults. However, following the introduction of Hib conjugate vaccines, the incidence of invasive Hib disease, including pneumonia, has declined dramatically in all countries where they are routinely used [10]. In this paper the current role of *H. influenzae* as a causative agent of pneumonia in children and adults will be reviewed. Relevant studies were identified by searching PubMed and Google Scholar for articles (published in English) including in their titles or abstracts the term ‘*Haemophilus* influenzae” combined with the any of the following terms: “pneumonia”, “community-acquired pneumonia”, “respiratorytract infection”, “epidemiology”, “burden”, “incidence”, “carriage”, “aetiology”, “mortality”, “morbidity”, “children”, “adults”, “vaccination”, “co-infection”, “respiratory viruses”, “interaction”, “microbiology”, “laboratory diagnostics”, or “taxonomy”. More citations were identified from references in these initial searches.

## 2. Microbiology

*Haemophilus* spp are pleiomorphic Gram-negative coccobacilli. *H. influenzae* is the major human pathogen in the group. Some strains of *H. influenzae* are encapsulated whilst others are non-encapsulated. There are six distinct antigenic serotypes of encapsulated *H. influenzae* designated a–f on the basis of their capsular polysaccharide. Hib has a polyribosyl ribitol phosphate capsule. NTHi strains cannot be serotyped by conventional type-specific antiserum agglutination. The different serotypes and NTHi vary in their pathogenic potential. Margaret Pittman [11] noted that most invasive disease was caused by Hib strains. whereas the majority of respiratory tract isolates were NTHi. The other capsulated serotypes are less common causes of infection.

The differential requirements for two accessory growth factors, X-factor (haemin) and/or V-factor (nicotinamide adenine dinucleotide), are important criteria for defining the *Haemophilus* spp. *H. influenzae* requires both X-factor and V-factor. Besides *H. influenzae*, a second X- and V-factor dependent species, *H. haemolyticus*, is frequently found in the upper respiratory tract and has long been regarded as a commensal. Until recently, microbiologists relied on the production of zones of β-haemolysis on horse blood agar to differentiate *H. haemolyticus* from NTHi. However, 10–40% of strains of *H. haemolyticus* are non-haemolytic [12] and several molecular methods have been proposed to accurately identify *H. haemolyticus* [13,14].

The capsular serotype of *H. influenzae* is traditionally determined by slide agglutination using type-specific antisera. This method is prone to misinterpretation, with problems of auto-agglutination, cross-reacting antisera, and observer variation. The definitive method of typing *H. influenzae* is capsular genotyping using a polymerase chain reaction (PCR)-based method [15]. Molecular typing will allow accurate differentiation between typeable and non-typeable strains. It will also, if the appropriate primers are used, distinguish true NTHi strains from *H. haemolyticus* and also from strains of *H. influenzae* that contain a complete or partial capsule locus. Strains with a partial capsule locus (capsule-deficient mutants, Hib-minus or Hib-strains) are unable to export polysaccharide to the cell surface and thus will appear to be NTHi because they are non-typeable by conventional means. NTHi and *H. haemolyticus* may also be differentiated by matrix-assisted laser desorption/ionisation time-of-flight mass spectrometry (MALDI-TOF) [14], which is increasingly becoming available in diagnostic clinical laboratories.

## 3. *H. influenzae* carriage

*H. influenzae* is only found in humans and colonises the nasopharynx and throat, and to a lesser extent the conjunctivae and genital tract. The respiratory tract is mainly colonised by *H. parainfluenzae* and NTHi. In developed countries, approximately 20% of infants will be colonised with NTHi in the first year of life and the rate increases over time with more than 50% of children aged 5 years carrying NTHi [16]. Carriage rates are much higher in children in developing countries and in indigenous communities, where NTHi carriage is established very soon after birth [17,18]. Carriage is a dynamic process and different patterns of colonisation are seen in the first two years of life, ranging from short-term colonisation with a single strain, to prolonged colonisation with one strain, to recurrent colonisation with different or multiple strains [18–20]. Children in day-care centres have both higher rates of colonisation with NTHi and more frequent transmission of strains compared to controls [21]. NTHi also colonise the upper respiratory tract of 20–30% of healthy adults [22,23]. NTHi are found in 30–40% of sputum cultures from patients with stable chronic obstructive pulmonary disease (COPD) [24,25] and approximately 50% during acute exacerbations of COPD [24–27]. These figures, based on traditional bacteriological culture, may be underestimates as studies using PCR have confirmed higher detection rates of NTHi in COPD [28]. The published data on the prevalence of lower respiratory tract colonisation with NTHI in healthy adults is based on small numbers of subjects but indicates that NTHi was present in 0–4% of subjects [25,27]. Children frequently carry multiple strains of NTHi [29].

Transmission occurs through the spread of respiratory droplets or contact with respiratory secretions. Adults with COPD may carry multiple strains of NTHi [30] and the acquisition of a new strain may be associated with an acute exacerbation [31]. There is also evidence that the combination of NTHi and rhinovirus infection can increase the severity of acute exacerbations [32]. Immunity to one strain of NTHi does not confer protection against colonisation by a different NTHi strain, nor does it prevent infection [33].

The carriage rate of capsulated strains of *H. influenzae* is much lower. In unvaccinated populations, 3–5% of infants and 8–12% of pre-school age children are colonised by Hib, with higher rates being found in children attending day-care centres [21], and among household members of a case of invasive Hib disease [34]. By the age of 5 years, almost all children in unvaccinated populations would have been colonised at some time by Hib.

### 3.1 Interaction between H. influenzae and respiratory viruses

In 1889–1992 an epidemic of influenza occurred in Western Europe. Richard Pfeiffer [35,36] examined sputum from patients suffering from influenza and described seeing almost pure cultures of a Gram-negative bacillus in the majority of samples. He postulated that *Bacillus influenzae* (Pfeiffer’s bacillus or *H. influenzae*) was the cause of influenza. In 1922, Kristensen [37] proposed that this organism was a secondary invader rather than the primary cause of influenza. In 1933, Smith, Andrewes, and Laidlaw [38] established that a virus caused influenza. A major cause of death in epidemics of viral influenza is superinfection with a bacterial pathogen, most commonly *S. pneumoniae*, but other common respiratory pathogens, including *H. influenzae, Staphylococcus aureus* and Group A streptococci have predominated in different pandemics orgeographical locations [39].

Exacerbations of COPD are often associated with viral and bacterial co-infection. The most common finding is co-infection with rhinovirus and *H. influenzae*, which is also associated with increased severity of the exacerbations [26,40].

The interaction between respiratory viruses and bacterial pathogens is multifactorial and complex [41,42]. Respiratory viral infections render the epithelial surface more susceptible to bacterial colonisation [43] by disrupting the epithelial barrier [44] whereby rhinovirus infection can facilitate paracellular migration of NTHi [45]. Furthermore, viruses impair mucociliary velocity and reduce bacterial clearance [44]. Viral infection also triggers a pro-inflammatory response, upregulating adhesion proteins in respiratory epithelial cells. For example, rhinovirus upregulates intracellular adhesion molecule 1 (ICAM-1), which facilitates not only its own invasion but also NTHi adhesion [46,47]. Respiratory viruses impair neutrophil function by decreasing the oxidative burst and enhancing neutrophil apoptosis, which increases susceptibility to bacterial superinfection [48]. In addition, viruses induce production of interferons (IFNs), IFN-α and IFN-β, which also impair neutrophil responses [49] and IFN-γ down-regulates macrophage activity thus impairing bacterial clearance [50]. Respiratory viruses also interact with Toll-like receptors (TLRs) desensitising the lung sentinel cells to bacterial ligands [51]. The interaction is not entirely one way since NTHi stimulates the expression of ICAM-1 and TLR-3 on human epithelial cells, facilitating rhinovirus entry [45].

## 4. Spectrum of infections caused by *H. influenzae*

*H. influenzae* is associated with two types of infection, invasive and non-invasive infections, which have distinctive epidemiologic profiles.

### 4.1. Invasive infections

Prior to the introduction of Hib conjugated vaccines, Hib was one of the most common causes of meningitis and pneumonia in infants and young children under the age of 5 years. In England and Wales, 90% of invasive *H. influenzae* infections were caused by Hib, 10% by NTHi, and less than 1% by other serotypes; 23% of NTHi infections occurred in children less than 5 years of age [52]. Bacteraemia with no obvious focus of infection was the commonest presentation of invasive NTHi infection (37%) followed by bacteraemic pneumonia (27%) and meningitis (12%).

The addition of Hib conjugate vaccines to infant immunisation programmes has resulted in a dramatic decline in invasive Hib infections in every country where they have been used, through a combination of direct and indirect (herd) protection [53,54]. Polysaccharide-protein conjugate vaccines have the capacity to reduce nasopharyngeal carriage of the target organism of the vaccine serotype/serogroup, which provides the basis of herd protection. Hib vaccination has no effect on the carriage of other serotypes of *H. influenzae* or NTHi, which can also, albeit less frequently, cause invasive infections including bacteraemic pneumonia [55].

### 4.2. Non-invasive infections

*H. influenzae* can cause a range of non-invasive infections of mucosal surfaces. NTHi is the predominant bacterial pathogen in respiratory tract infections in both children and adults: otitis media in infants and young children, sinusitis in older children and adults, non-bacteraemic pneumonia in elderly adults, and acute exacerbations of COPD in adults [55].

## 5. Determining the aetiology of community-acquired pneumonia (CAP)

There are a number of challenges facing any review of the aetiology of CAP. Pneumonia can be defined as an inflammatory response to infection in the alveoli and distal airways. Although this can be confirmed histologically, the clinical and radiological presentations are extremely diverse. The Pneumococcal Vaccines Accelerated Development and Introduction Plan (Pneumo-ADIP) developed a case definition for childhood pneumonia, based on cough, respiratory difficulty, and tachypnoea [56]; however, many published studies use different case definitions, making comparisons difficult.

Since NTHi are such common members of the upper respiratory tract flora, and mucosal infections are frequently polymicrobial, differentiating colonising commensals from the causative pathogen(s) is challenging. In the case of lower respiratory tract infections, including pneumonia, these uncertainties are compounded by difficulties in obtaining specimens that accurately reflect the microbiology of the lower respiratory tract. Sputum cultures are generally contaminated with upper respiratory tract flora and results should be carefully evaluated by considering the quality of the specimen. A commonly used approach is the molecular analysis of the cellular composition of sputum [57] where any sputum that contains >25 squamous epithelial cells/low power field in a 100x magnification of a Gram-stained smear of the sample is discarded. Another approach is quantitative cultures of expectorated sputum, where the expected threshold for significance of any potential pathogen is ≥10^6^ colony forming units (cfu)/ml [58].

Young children cannot produce adequate sputum samples unless sputum is induced with inhalations of hypertonic saline and nasopharyngeal aspiration. Although sputum induction appears to produce good quality samples for microbiological analysis, the procedure is not recommended for routine use as children generally find it unpleasant [59].

Broncho-alveolar lavage (BAL) is a more reliable sample as it is less likely to be contaminated by organisms in the upper airway. Quantitative culture of BAL specimens or samples collected through double-lumen brush catheters, using a threshold of ≥10^4^ cfu/ml, can improve the determination of the aetiology of non-bacteraemic CAP [60,61]. A transthoracic needle aspiration or diagnostic lung tap will assist in determining the aetiology in both children and adults [62–66], but in practice this procedure is impractical in the majority of cases of CAP, despite being a generally safe procedure with a low complication rate [65].

Serological tests may be used to detect a range of viruses and atypical pathogens but are often of limited use in clinical practice due to the requirement for acute and convalescent sera. *S. pneumoniae* and *L. pneumophila* antigen may be detected in urine and provide a diagnosis of CAP, though for the pneumococcus this test is unreliable in young children as a positive result may simply reflect the high load of pneumococci carried in the nasopharynx. No such test is currently available for *H. influenzae*, and would suffer from the same drawbacks as pneumococcal urinary antigen detection.

Quantitative real-time PCR (qRT-PCR) to detect *S. pneumoniae* and *H. influenzae* genes in serum or whole blood is a possible approach to the diagnosis of CAP. Rello et al [67] used qRT-PCR to detect pneumococcal DNA (*lytA*) in the blood of 62% of adults with confirmed or probable pneumococcal CAP. Blood cultures were positive in only 37% of the patients. This study also reported a correlation between bacterial DNA load and the risk of septic shock or death. Patients who had been pre-treated with antibiotics were excluded. Other groups included such patients in their studies with similar findings [68,69]. These studies were in adults. In paediatric CAP there is a concern that high rates of nasopharyngeal carriage may result in false positive results. Lai et al [70] demonstrated that dense upper respiratory tract colonisation with *S. pneumoniae* and *H. influenzae* did not produce false positive results when qRT-PCR was performed to detect *S. pneumoniae lytA* and *H. influenzae glpQ* genes in serum samples from Indigenous Australian children with a bacterial load of up to 10^6^ cfu/ml in matched nasopharyngeal swabs. This was a pilot study using spiked sera. Its applicability in clinical samples from children with suspected CAP is awaited. There is a need for standardisation of methodologies, “cutoff” values, and gene targets if meaningful comparisons are to be made between published studies [71,72].

The use of new methods for identifying the cause of CAP has produced evidence that many cases of CAP may be the result of mixed infections with either viral and bacterial or mixed bacterial aetiology in both adults and children [73–76]. The presumption in the case of mixed viral-bacterial aetiology is that a preceding upper respiratory tract viral infection predisposes the patient to a supervening bacterial infection.

An assay for specific biomarkers, such as procalcitonin, in conjunction with qRT-PCR has been proposed to differentiate viral from bacterial pneumonia [77]. However, since up to 30% of children hospitalised with CAP have co-infection with viruses and bacteria [73], this may be of limited use as a diagnostic test.

Slupsky et al [78,79] reported that nuclear magnetic resonance metabolomics may offer a way of differentiating pneumococcal pneumonia from other viral and bacterial causes of pneumonia, and may be a way of identifying children with severe pneumonia [80]. Since specific microbes produce unique urinary metabolites in the urine [81], this approach may be sufficiently specific to identify *H. influenzae* CAP. Whether this approach can be used to distinguish NTHi from other types of *H. influenzae* as a cause of pneumonia is unclear.

Several authoritative guidelines on the management and treatment of CAP state that it is not necessary to perform any microbiological investigations on cases of mild CAP in adults and children that do not require hospitalisation [82–85]. The rationale for this advice is that the investigations will not alter the management of the patient in the majority of cases. If the patient fails to respond to empirical antimicrobial therapy or requires hospitalisation, then the guidelines do recommend investigations. If the patient has already received broad-spectrum antimicrobial therapy, the likelihood of obtaining accurate information on the microbiological aetiology of the CAP is markedly reduced. The result of this is that the microbiological aetiology of non-bacteraemic CAP is ill-defined. The consequences of such an approach are not only that the causative pathogen(s) are not identified in the individual patient, but also that it hampers any assessment of the impact of vaccinations and may result in unnecessary antimicrobial therapy.

Radiological signs of alveolar consolidation are considered the most specific indicator of bacterial pneumonia [86,87]. To assess the reliability of paediatric chest radiograph interpretation, three paediatric sub-specialists (infectious diseases, radiology and respiratory medicine) reviewed 3,033 chest radiographs taken from children aged <5 years presenting with an acute febrile illness. The radiologist was most likely (21.3%) and respiratory physician least likely (13.7%) to diagnose consolidation. The overall percentage agreement for pairs of readers was 85–90%. However, chance corrected agreement between the readers was moderate, with kappa scores of 0.4–0.6. The estimated sensitivity ranged from 0.71 to 0.81 across readers, and specificity 0.91 to 0.98 [88]. However, as with microbiological investigations for pneumonia, many cases of CAP will be treated in the community and will not receive a chest radiograph.

If bacteraemic pneumonia is suspected, blood cultures should be taken. However, the sensitivity of blood cultures is generally low, especially when antimicrobial chemotherapy has already been initiated and the yield of significant pathogens from blood cultures ranges from 13–27% in children with complicated CAP, but is less than 5% in those with mild or moderate CAP [89,90]. *H. influenzae* is a highly fastidious organism and the recovery of this bacterium is highly dependent on both the blood culture broth and the media that are used for subculturing any potentially positive blood cultures [91]. Delay in incubating the blood cultures and the presence of antimicrobials in the blood will considerably reduce the chance of recovering *H. influenzae*. Commercial blood culture systems provide quality assured blood culture broths that do support the growth of *H. influenzae*, but it is also important to use appropriate media that contain adequate concentrations of X- and V-factors when subculturing any bottles that signal “positive”. Blood cultures may therefore significantly underestimate the presence of pathogens, including *H. influenzae*, in the lower respiratory tract. An alternative approach to conventional blood cultures is the detection of bacterial DNA by RT-PCR from dried blood spot samples placed on specialised filter papers, such as FTA® Elute (GE Healthcare Life Sciences, USA) [92].

## 6. *H. influenzae* as a cause of community-acquired pneumonia (CAP)

Hib, other capsulated serotypes, and NTHi can all cause pneumonia, which may be bacteraemic or non-bacteraemic in both children and adults.

### 6.1. Hib

Hib was a major cause of bacterial pneumonia in children prior to the introduction of Hib conjugate vaccines. Evidence for this is provided from surveys of bacteraemic infections in children, lung aspiration studies, and vaccine probe studies [93]. Vaccine probe studies from The Gambia indicated approximately 20% of severe pneumonia cases in young children were caused by Hib [94]. The widespread use of Hib conjugate vaccines has resulted in a decline in the number of cases of Hib disease, including bacteraemic pneumonia, wherever the vaccine has been implemented. However, invasive Hib infections do still occur. For example, prospective, enhanced population-based surveillance of invasive *H. influenzae* disease has been undertaken in England and Wales since 1990 [95]. Prior to the introduction of the Hib conjugate vaccine, invasive *H. influenzae* disease was predominantly caused by Hib (90%) with 90% of cases occurring in young children. Meningitis was the most common presentation (56%) of Hib infection, with bacteraemic pneumonia accounting for 5% of cases overall ranging from 3% of the cases in children <15 years of age to 31% in adults. NTHI was responsible for 10% of invasive *H. influenzae* disease, and bacteraemic pneumonia was the clinical presentation in 13% of children <15 years of age and 34% of adults [52].

**Figure 1:**
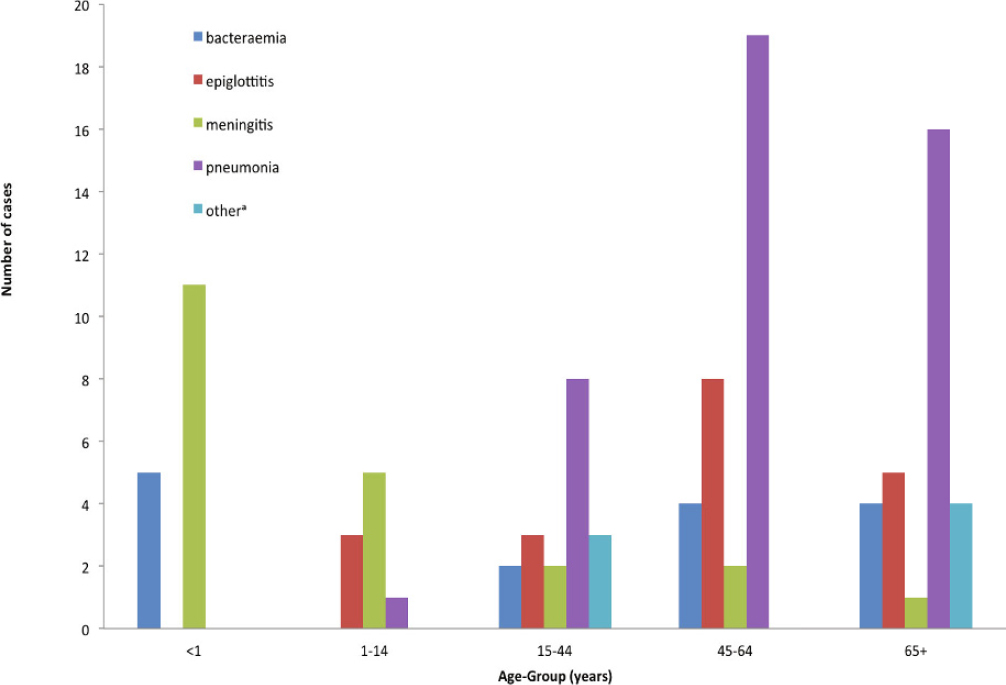
Cases of invasive *Haemophilus influenzae* type b (Hib) disease in England and Wales, 2009–2012, by age and clinical presentation (Based on data from the Public Health England surveillance of invasive *H. influenzae* infections. Available from https://doi.org/www.gov.uk/government/collections/Haemophilus-influenzae-guidance-data-and-analysis) ^a^cholecystitis, discitis, endocarditis, and genitourinary infections

The Hib conjugate vaccine was incorporated into the UK infant immunisation schedule in 1992. Since that time, the incidence of invasive Hib disease in children <5 years of age has fallen from 35.5 per 100,000 in 1990 to 0.06 per 100,000 in 2012 [96]. Concomitant with the changing incidence, the age distribution of invasive Hib disease and its clinical presentation has altered. Of 106 cases of invasive Hib disease occurring over a 4-year period (2009–2012), 73% occurred in adults (Figure 1), often with pre-existing co-morbidities, and 56% of the adult cases presented as a bacteraemic pneumonia [96].

Invasive Hib disease is principally found in adults, often with pre-existing medical conditions who often present with pneumonia [96]. Hib remains a leading cause of pneumonia in children in countries that have not yet introduced the Hib conjugate vaccine [97].

### 6.2. NTHi and Non-type b capsulated H. influenzae

A major concern following the introduction of routine immunisation against Hib was that as conjugate vaccines also prevent carriage, other *H. influenzae* serotypes or NTHi might fill the ecological niche in the nasopharynx and, consequently, lead to increased risk of invasive disease [98]. These concerns have so far remained unfounded, with no conclusive evidence for replacement disease. However, because of the success of the Hib conjugate vaccine, NTHI has become by far the most common cause of invasive and non-invasive *H. influenzae* infections across all age groups in countries with established Hib vaccination programmes [98].

#### 6.2.1 NTHi

NTHi are a recognised cause of both bacteraemic and non-bacteraemic pneumonia in both children and adults, though the data on the relative importance of NTHi in the microbial aetiology of paediatric pneumonia are limited and to some extent conflicting [99]. Silverman et al [62] investigated 88 Nigerian children (aged 4 months to 8 years) with severe, untreated, acute pneumonia. Using standard bacteriological culture of blood and nasopharyngeal secretions plus needle aspiration of consolidated lung tissue, they identified *H. influenzae* in 10/88 (14.3%) lung aspirates. The isolates were all negative when tested by counter-current immunolectrophoresis with Hib antiserum. This confirmed that the isolates were not Hib and it is reasonable to assume that some, if not all, of the isolates were NTHi. Studies from Papua New Guinea, The Gambia, and Pakistan in the 1980s suggested that, in these communities, NTHi was a major cause of pneumonia in children [100–104]. Using blood cultures and lung aspiration, Shann and colleagues [100] studied the aetiology of pneumonia in 83 children admitted to hospital in Goroka, Papua New Guinea. *H. influenzae* was the predominant pathogen, being found in 33/83 (40%) of the cases. Pneumococci were identified in 34% and *M. catarrhalis* in 11% of the cases [100]. Of the haemophili typed, Hib accounted for 19% (6/32) of infections and 56% (18/32) were NTHi. Co-detection of *H. influenzae* and *S. pneumoniae* occurred in 22% of the children. Wall et al [102] reported a study of lung aspirates and blood collected from 64 Gambian patients with pneumonia, including 51 children aged <10 years. They found *S. pneumoniae* in 51% (26/51) of children aged <10 years of age and *H. influenzae* in 23% (11/49) of the children aged <5 years. Of the haemophilus isolates, 31% (4/13) were NTHi. A study of acute lower respiratory tract infections in children from Pakistan [103,104] identified *H. influenzae* and *S. pneumoniae* in 10%, respectively, of 1,331 cases of pneumonia, and NTHi accounted for 32% (34/105) of the haemophilus isolates isolated from blood cultures.

A more recent study of 55 children (aged 2–59 months) with pneumonia from The Gambia [105] reported improved detection rates when using PCR as opposed to culture for both *S. pneumoniae* (25% [14/56] versus 91% [48/53]) and *H. influenzae* (5% [3/56] versus 23% [12/53]). Yin et al [106] prospectively studied 1,158 children admitted to hospital over three 1-year periods in Singapore with community-acquired LRTI. Non-type b *H. influenzae* (presumably NTHi) was identified as the causative agent in 9% (101/1,158) of cases. NTHi was found in 50/479 (10.4%) children aged <1 year, in 47/532 (8.8%) children aged 1–5 years, and in 4/147 (2.7%) children aged >5 years. Rahman et al [107] examined blood cultures collected from 1,493 children aged <5 years admitted with pneumonia to three hospitals in Dhaka, Bangladesh over the period 1999–2003. They identified 25 cases of bacteraemic CAP caused by *H. influenzae*: 15 Hib, 6 NTHi, 3 serotype c (Hic), and 1 serotype d (Hid). The majority of these infections occurred in infants aged between 4 and 12 months.

In a retrospective study of CAP aetiology [61], flexible bronchoscopy with BAL was performed on 127 children with acute non-responsive CAP and 123 children with recurrent CAP. The children ranged in age from 1 month to 15 years (median age 33 months) and were otherwise healthy. Children with severe or chronic disease (cystic fibrosis, asplenia, bronchopulomonary dysplasia, primary ciliary disease, immunodeficiency, and tuberculosis) were excluded from the analysis. An infectious agent was identified in 76.0% (190/250) of cases, and aerobic bacteria were isolated in 51.2% (128/250) of the infections: namely *H. influenzae, M. catarrhalis* and *S. pneumoniae* in 75.0% (96/128), 28.9% (37/128) and 13.3% (17/128) of cases, respectively. Almost all of the *H. influenzae* isolates (97.9% [94/96]) were NTHi. The identification of NTHi was confirmed by conventional serotyping and PCR and the strains were also distinguished from *H. haemolyticus*. NTHi were identified in 26.0% (33/127) of cases of non-responsive CAP and 51.2% (63/123) of cases of recurrent CAP. These studies provide details of the serotyping of the *H. influenzae* isolates (Table 1). Unfortunately, most of the other studies in the literature do not give any data on serotyping and so it is difficult to know what proportion of infections were due to NTHi, Hib, or other capsulated serotypes. A systematic review and meta-analysis of CAP in children in Latin America and the Caribbean [108] found that *H. influenzae* ranked first as a cause of CAP in infants aged 0–23 months. The authors noted that all of the included studies took place before 2000, at a time when most Latin American countries had not yet introduced the Hib conjugate vaccine, and so they presumed Hib probably caused most of these infections. However, no data on serotyping was given in the paper. A recent review of the global epidemiology and aetiology of childhood pneumonia in 2010 [2], which summarised the causative pathogens in childhood CAP in 192 countries, gave data for *H. influenzae* CAP with no breakdown of the data by serotype. It is to be hoped that future studies, such as the large ongoing project funded by the Gates Foundation (the Pneumonia Etiology Research for Child Health [PERCH] study) [109] will be able, by using molecular and conventional techniques, to determine the relative importance of Hib, NTHi and non-type b capsulated serotypes of *H. influenzae* as causative agents in childhood CAP.

**Table 1:** Summary of published studies of paediatric community-acquired pneumonia (CAP) in which non-typeable *Haemophilus influenzae* (NTHi) was identified as a significant pathogen

Authors	Country	Study details	Number of cases	Age of children	Year of study	Assay	Diagnostic criteria	Number of positive cultures (%)	*H. influenzae* isolated (%)	NTHi: proportion of total *H. influenzae* isolates (%)	Virology	Hib vaccine introduced
Silverman et al [62]	Nigeria	Children with severe, untreated, acute pneumonia	88	4 months to 8 years	1977	Lung aspirate	Growth	70/88 (80%)	10/88 (11%)	All CIE negative ie not Hib	no	no
						Blood culture	Growth	4/36 (11%)	0/36 (0%)			
						Sera	CIE for pneumococci and Hib	9/45 (20%)	0/45 (0%)			
Shann et al [100]	Papua New Guinea	Children hospitalised with pneumonia	83	<5 years	1978–1988	Blood culture	Growth	51/83 (61%)	19/83 (23%)^a^	4/19 (21%)	yes	no
						Lung aspirate	Growth		33/83 (40%)^a^	18/32 (56%)		
Wall et al [102]	The Gambia	Children and adults with pneumonia	64	51 aged <16 years	1982–1984	Blood culture	Growth	14/51 children (28%); 2/13 adults (15%)	11/49 (23%) children <5 years; 2/15	0/9 (0%)	no	no
						Lung aspirate	Growth	29/51 children (57%); 7/13 adults (54%)	(13%) older patients^b^	4/13 (31%)		
						Sera	CIE for Hib	2/64 (3%)	0/6 (0%)	0/2 (0%)		
Ghafoor et al^c^[103]	Pakistan	Children hospitalised with pneumonia	1,331	<5 years	1986–1988	Blood culture	Growth	276/1,331 (21%)	144/1,331 (11%)	34/105 (32%)	yes	no
Yin et al [106]	Singapore	Children with pneumonia	1,158	<15 years (median age 1.37 years)	1988, 1995, 1999	Blood culture	Growth	671/1,158 (58%)	0/8 (0%)		yes	no
						Pleural fluid	Growth		0/15 (0%)			
						Sputum	Growth		101/1,158 (9%)	101/101 (100%)^d^		
Rahman et al [12]	Bangladesh	Children hospitalised with pneumonia	1,493	<5 years	1999–2003	Blood culture	Growth	25/1,493 (1.7%)	25/1,493 (1.7%)	6/25 (24%)^e^	no	no
De Schutter et al [61]	Belgium	Children with acute nonresponsive or recurrent CAP	250	1 month to 15 years (median age 33 months)	2005–2007	BAL	Growth with quantitative culture (≥10^4^ cfu/ml) and PCR	190/250 (76%)	33/127 (26%) in NR13AP; 63/123 (51%) in Rec-CAP	94/96 (98%)^f^	yes	yes
Howie et al [105]	The Gambia	Children with severe pneumonia	55	2 months to <5 years	2007–2008	Lung aspirates (*n*=47) and pleural aspirates	Growth	21/56 (38%)^g^	3/56 (5%)^g^	3/3 (100%)	yes	yes
							PCR	28/53 (53%)	12/53 (23%)	3/4 (75%)^h^		

BAL, broncho-alveolar lavage; CIE, counter-current Immunoelectrophoresis; PCR, polymerase chain reaction; NR-CAP, non-responding CAP; Rec-CAP, recurrent CAP; Spn, *Streptococcus pneumoniae*; cfu, colony forming units; Hia, *Haemophilus infuenzae* type a; Hib, *Haemophilus infuenzae* type b; Hic, *Haemophilus infuenzae* type c; Hid, *Haemophilus infuenzae* type d; Hie, *Haemophilus infuenzae* type e; Hif, *Haemophilus infuenzae* type f; *n*, number

^a^32/42 (76%) *H. influenzae* isolates serotyped. Other serotypes included 1 Hia, 6 Hib, 3 Hic, 2 Hid, 1 Hie and 1 Hif. *H. influenzae* frequently co-cultured with Spn. Spn isolated from 28/83 (34%) children

^b^*H. influenzae* isolate serotypes included 2 Hia (from lung) and 7 Hib (Of these, 3 from lung)

^c^See also Weinberg et al [104] for more details of this study

^d^NTHi only recovered from sputum samples which were from older children and only assessed if there were <25 epithelial cells/high power field

^e^Other serotypes included 15 Hib, 3 Hic and 1 Hid

^f^2 isolates lost for typing

^g^56 specimens were cultured. NTHi and Spn co-cultured in 21% (11/53) of cases

^h^1/4 (25%) isolates was Hib

Several studies have examined serological evidence of a pathogenic role for NTHi in lower respiratory tract infections [110–114]. Claesson and colleagues [110,111] measured the serum antibody responses to NTHi in 38 children (mean age 4.4 years) with radiologically proven pneumonia in whom NTHi in the nasopharynx was the only potential causative pathogen, and in 38 age, sex-matched children with radiologically proven pneumonia in whom NTHi was not isolated. Sixteen out of 38 (42%) cases of the NTHi group had significant antibody responses (IgG, IgM, or both) to a preparation of NTHi outer membrane proteins, compared to 2/38 (5.2%) of the control group (p < 0.001, z test for comparison of 2 proportions). Korppi et al [112] studied 449 children aged <15 years hospitalised for respiratory tract infections. Serological evidence of NTHi infection was demonstrated in 6% of the children. An infection focus was demonstrated in 25 children with NTHi infection, and in 10 cases this was pneumonia. The authors concluded that NTHi is a genuine respiratory tract pathogen in children, but NTHi infections appear to be secondary to preceding viral or bacterial infections in children. Juven et al [73] carried out a 3-year prospective study of 254 children with CAP. NTHi was identified in 22 cases (9%), frequently in association with evidence of a viral infection.

In adults the role of NTHi as a cause of pneumonia is well-recognised [115]. Everett et al [116] reported 18 cases of *H. influenzae* pneumonia in adults aged 17–87 years, of whom the majority were >50 years of age. The patients were admitted to an Army Medical Center over a 3-year period (1973–1976) but the paper does not state the total number of cases (i.e. the denominator) of pneumonia studied. Eleven of the patients had comorbidities. Transtracheal aspirates and blood cultures were performed. Unfortunately only 5 of the isolates were serotyped, but 4/5 were NTHi. Gotfried [117] reported on 610 cases of CAP in adults ≥18 years seen in the primary care setting in the USA. Sputum samples, either expectorated or induced were collected and mailed to a central laboratory for processing. Sputum samples with >25 neutrophils and <10 squamous epithelial cells per low power field were deemed suitable for microbiological culture. Of the 610 samples submitted, 204 yielded positive cultures and *H. influenzae* was the most prevalent (38%) organism identified. The strains of *H. influenzae* were not typed but it is probable that the majority were NTHi. More than 50% of the patients were smokers or had a history of COPD. Another study conducted in Papua New Guinea prospectively examined 170 adult patients with acute CAP. Of these, *H. influenzae* was identified as the sole pathogen in 15 (9%) patients: 7 Hib, 4 serotype a (Hia), 1 Hic, and 3 NTHi [118]. Chronic lung disease was more common in patients with *H. influenzae* pneumonia than CAP due to other organisms. A further report from these investigators [119] identified Gram-negative bacilli, including *H. influenzae*, in 26% of the 90 culture positive percutaneous lung aspirates performed on 144 adult patients with CAP. However, no details on the serotypes of the strains are reported. Janssens and Krause [120] reviewed pneumonia in the elderly and noted that *H. influenzae* is amongst the most frequently reported pathogens in older patients with CAP (up to 14%) and was identified in 7% (4/57) of elderly patients with severe CAP requiring admission to an intensive care unit [121]. NTHi is frequently linked to acute exacerbations of COPD and bronchiectasis and should be regarded as a potential pathogen in these patients [122].

Campos et al [123] reported on invasive *H. influenzae* infections in the Madrid, Spain, area over a 2-year period (Jan 1999–Dec 2000) at a time of widespread use of the Hib conjugate vaccine. Ofthe *H. influenzae* isolates, 67/91 (73.6%) were NTHi and 16.5%, 6.6%, and 3.3% were types b, f (Hif), and e (Hie), respectively. There were 23 cases of pneumonia and 79% of these were due to NTHi. Pneumonia was more prevalent in adults (>14 years) than in children (<14 years): 25.9% versus 7.1% (*p* = 0.03).

Berndsen, Erlendsdóttir, and Gottfredsson [124] reported on population based surveillance of invasive *H. influenzae* infections in Iceland from 1983 to 2008. Since the introduction of the Hib conjugate vaccine, NTHi has become the most commonly isolated *H. influenzae* (40/59 isolates) from patients with pneumonia or bacteraemia, followed by Hif (14/59 isolates) which principally caused bacteraemic pneumonia. Underlying diseases were more common in both children and adults with NTHi infection compared to invasive Hib disease.

**Figure 2:**
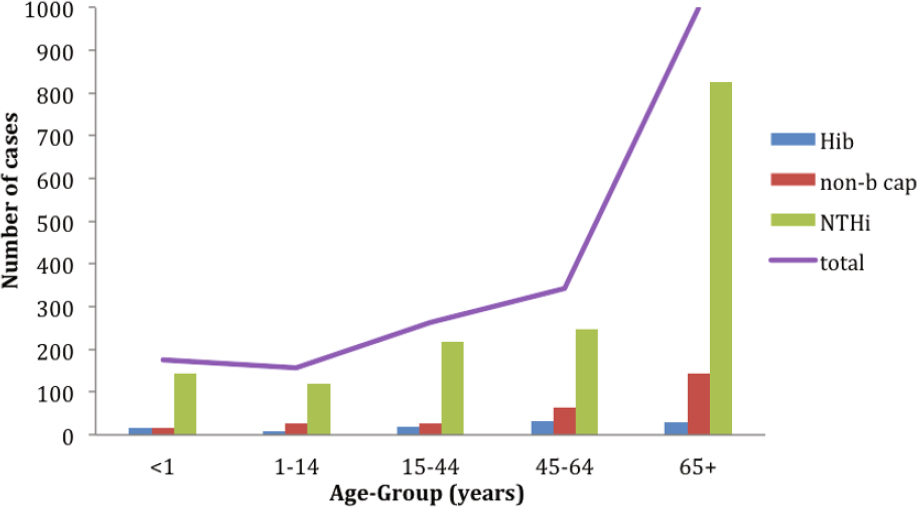
Cases of bacteraemic pneumonia in England and Wales, 2009–2012, by age group and serotype (Based on data from the Public Health England surveillance of Invasive *Haemophilus influenzae* infections. Available from https://doi.org/www.gov.uk/government/collections/Haemophilus-influenzae-guidance-data-and-analysis)

In England and Wales, the most common clinical presentation of invasive NTHi disease overall is pneumonia, which increases with age and occurs mainly in older adults, many of whom have underlying respiratory tract comorbidities [55] (Figure 2).

More recently, Torres et al [125] summarised the data available from 33 studies, published between 2005 and 2012, on the aetiology of CAP and/or antibiotic treatment in adults across Europe. Microbiological methods used were reported in 22 of the studies and were similar across all studies. The methods used included assessment of blood, sputum, urine (urinary antigen tests specifically for the detection of pneumococci and legionella) and pleural fluid samples, and less commonly, tracheobronchial, bronchoalveolar, transthoracic, and nasopharyngeal samples. Blood cultures were also performed in all 22 studies. *S. pneumoniae* was the most commonly isolated pathogen, being identified in 12.0–85.0% of patients within 19 studies, whilst *H. influenzae* was isolated in up to 29.4% of patients with 15 studies. *H. influenzae* (<65 years: 4.1–6.4%; ≥65 years: 2.9–29.4%) and *S. pneumoniae* (<65 years: 20.9–28.0%; ≥65 years: 19.9–85.6%) were isolated more frequently in adults over the age of 65 years. The aetiology of CAP was similar in patients with and without COPD. Since these studies were conducted at a time when Hib immunisation of infants had been introduced across all of the participating countries, it is highly probable that these are cases of NTHi CAP, but again no details of serotyping results are given.

Peto et al [126] systematically reviewed 48 studies of adult CAP in Asia, excluding the Middle East, published between 1990 and 2012. From a total population of 10,423, the overall rate of *H. influenzae* (serotype unspecified) CAP in Asia was 6.9% with rates varying from 19% in the Philippines, 10% in Japan and 9% in China, to 1% in India and South Korea. In most of the studies reviewed, 35–70% of cases had no pathogen identified.

Impaired respiratory defense mechanisms may play a role in the pathogenesis of NTHi pneumonia in adults. Approximately 50% of healthy adults aspirate small amounts of oropharyngeal secretions during sleep, but the lower respiratory tract is protected from recurrent infections by effective mucociliary clearance, humoral, and cellular immune mechanisms, and coughing [120]. In elderly patients these defense mechanisms will be to an extent impaired and silent aspiration is an important factor in the development of CAP.

#### 6.2.2 Non-type b capsulated H. influenzae

**Figure 3:**
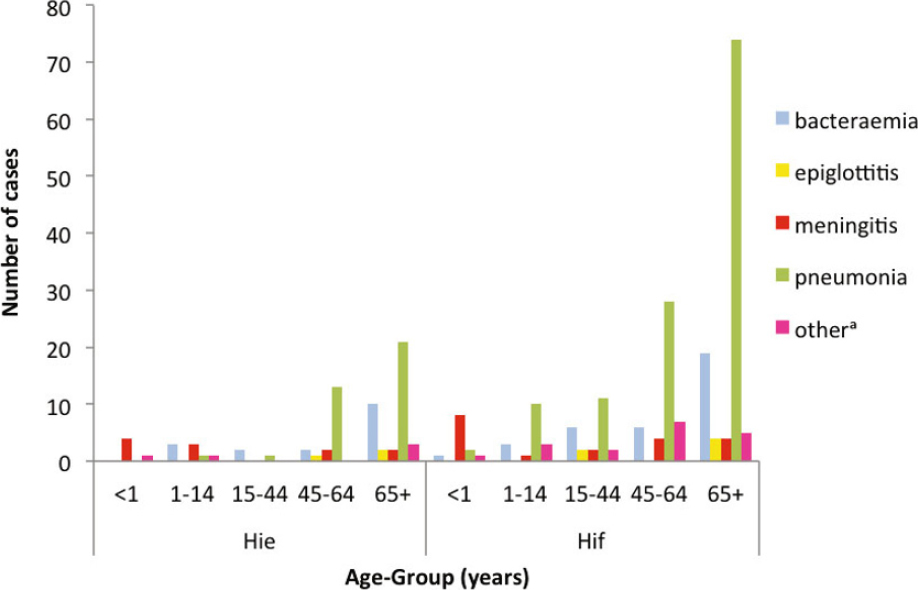
Cases of *Haemophilus influenzae* type e (Hie) and *H. influenzae* type f (Hif) bacteraemia in England and Wales, 2009–2012, by age group and clinical presentation (Based on data from the Public Health England surveillance of invasive *H. influenzae* infections. Available from https://doi.org/www.gov.uk/government/collections/Haemophilus-influenzae-guidance-data-and-analysis) ^a^includes cases of invasive *H. influenzae* disease which presented with focal infections not listed above including cellulitis, cholecystitis, discitis, endocarditis, osteoarthritis, septic arthritis and genitourinary infections

In countries where long-term surveillance of invasive *H. influenzae* disease has been conducted pre- and post-Hib conjugate vaccine implementation, there has been a small but steady increase in the incidence of invasive *H. influenzae* infections due to other serotypes of *H. influenzae*. The majority of these infections are due to NTHi (see Section 6.2.1), but there has also been a small increase in non-type b capsulated strains. For example, in England and Wales during the period 2001 to 2010, a total of 1,275 invasive *H. influenzae* infections were reported, including 715 (56.1%) NTHi, 69 (5.4%) Hib, 99 (7.8%) Hif, and 33 (2.6%) Hie [127]. In 2001 there were 8 cases of invasive Hie infection, giving an adjusted annual estimated incidence of 0.021 (95% CI 0.009–0.040) per 100,000 population and 27 cases of Hif infection with an adjusted annual estimated incidence of 0.069 (95% CI 0.046–0.101) per 100,000 population. In 2010, there were 15 cases of Hie invasive infection, with an adjusted annual estimated incidence of 0.036 (95% CI 0.020–0.060) per 100,000 population and 52 cases of Hif infection, with an adjusted annual estimated incidence of 0.126 (95% CI 0.094–0.166) per 100,000 population. Ten cases (7 Hif, 3 Hie) occurred in the first year of life and none of these infants presented with pneumonia. There were 13 cases in children aged 1–4 years (8 Hif, 5 Hie), of whom 8 had underlying comorbidities. Four of these children had pneumonia. Over the same time period the number of cases of invasive NTHi infection increased from 220 in 2001 (adjusted annual estimated incidence 0.564 [95% CI 0.492–0.644] per 100,000 population) to 303 cases in 2010 (adjusted annual estimated incidence 0.736 [95% CI 0.656–0.824] per 100,000 population). Of the 132 Hif infections, 53 occurred in adults >15 years of age, and 52 occurred in adults aged ≥65 years (based on data from the Public Health England surveillance of invasive *H. influenzae* infections. Available from https://doi.org/www.gov.uk/government/collections/Haemophilus-influenzae-guidance-data-and-analysis). Pneumonia was the commonest presentation for both Hif and Hie, occurring in 53/83 (63.9%) adults with invasive Hif disease and 16/24 (66.7%) adults with invasive Hie disease (Figure 3). The majority of the patients had at least one comorbidity: the percentage with comorbidities rising from 70% for those aged 15–44 years to 97% for those aged >65 years. The Case Fatality Ratio (CRF) for Hif pneumonia was 11.5% and 41.2% for Hie pneumonia. Although Hie infections were less common than Hif, they appear to be more virulent with a higher infection-attributable case fatality rate even after adjustment for age and comorbidities. Invasive Hif and Hie disease in England and Wales appears to share many of the features of invasive NTHi disease, in that it tends to occur in older patients with underlying co-morbidities, commonly presents as pneumonia, and has a higher CFR compared to Hib infections in children [127,128]. Resman et al [129] reported a similar increase in both NTHi and Hif invasive infections in southern Sweden. In a retrospective study over the period 1997 to 2009, they identified 410 cases of invasive *H. influenzae* disease comprising 29 Hib, 44 Hif, 1 Hie, and 191 NTHi. NTHi was predominant in all age groups including children <5 years of age. In 1998, the authors identified 8 cases of invasive NTHi disease and 2 cases of invasive Hif infection. In 2007, there were 35 NTHi invasive infections and 16 Hif invasive infections. A statistically significant increase in invasive disease by NTHi (Constant = 0.079, 95% CI 0.046–0.111; *p* < 0.001) and Hif (Constant = 0.023, 95% CI 0.003–0.043, *p* < 0.025) was observed, whereas the incidence of Hib disease was unchanged during the study period. Of the invasive non-type b infections, 70% presented with pneumonia and 59% of invasive Hif infections met the definition of severe sepsis or septic shock. The median age of Hif patients was 60 years and the CFR for Hif infections was 14%. Interestingly, in this series, 68% of invasive Hif infections occurred in individuals without any pre-existing co-morbidities.

Hia has also been recognised as an important cause of invasive disease in some parts of the world [130], notably in indigenous populations in Canada and USA. The range of clinical presentations, including bacteraemic pneumonia, is analogous to that of Hib in the pre-vaccine era, with the exception that Hia does not appear to cause epiglottitis. As with Hie and Hif, adults are more likely to present with pneumonia, whereas children generally present with meningitis [131]. Interestingly, there is as yet no evidence of an increase in invasive disease due to non-type b serotypes or NTHi in Australian Indigenous children [132].

## 7. Discussion

There is good evidence that all serotypes of *H. influenzae* are respiratory pathogens and can cause CAP. NTHi are recognised as a common cause of CAP in adults, particularly those with underlying respiratory disease. The quality of data supporting a role of NTHi as a cause of paediatric CAP is more limited [29,133]. Data showed a clear role for NTHi in paediatric pneumonia in Papua New Guinea and The Gambia in the 1980s. However, this finding was generally not replicated in contemporaneous studies from the developed world. Although, De Schutter et al [61] have clearly demonstrated that with good quality specimens and appropriate conventional and molecular microbiological techniques, NTHi is indeed a significant cause of CAP in young European children.

There are a number of possible reasons for the disparity in the published studies. The generally held view is that NTHi is an uncommon cause of pneumonia, except in adults with COPD. It is therefore possible that if NTHi are isolated from respiratory samples they may simply be dismissed as contaminants rather than the organism causing the infection. The routine bacteriological and blood culture methods used may be inappropriate for the recovery of a fastidious organism like *H. influenzae*, and compared to NTHi, is less likely to invade the blood stream. It is of note that the studies from Papua New Guinea, Pakistan and The Gambia [100–104], which employed lung aspirates, did detect NTHi as a significant cause of paediatric pneumonia.

Many of the published studies do not give details of serotyping of strains of *H. influenzae*, and prior to the introduction of Hib conjugate vaccines, Haemophilus pneumonia was probably assumed to be due to Hib. Conventional serotyping is less reliable than PCR-based molecular typing. NTHi invade the blood stream less readily than Hib and so would represent a small proportion in any series of bacteraemic pneumonias. Non-bacteraemic pneumonia is a mucosal infection which is often polymicrobial, with a bacterial infection following a viral infection. Therefore, the role of NTHi in non-bacteraemic pneumonia could be under-reported or overlooked. NTHi are well-recognised as a major cause of upper respiratory tract infections including otitis media and sinusitis. It therefore seems highly probable that this organism is capable of causing lower respiratory tract infections, possibly by contiguous spread from the upper airway. A preceding viral upper respiratory tract infection may facilitate the development of lower airways infection, including CAP (see Section 3.1). There are no data indicating whether different strains of NTHi are involved in asymptomatic colonisation and infection, but whole genome analysis of carriage strains and strains isolated from lower respiratory tract infections may identify adaptive features that could be applied as diagnostic tools.

In order to determine the extent to which *H. influenzae*, including NTHi, is a significant cause of bacteraemic and non-bacteraemic paediatric and adult CAP, a systematic study should ideally be undertaken using the most appropriate sampling techniques and applying the best methods of testing to those samples. In children where BAL or lung taps are not performed and who cannot expectorate sputum, it may be that an algorithmic approach is necessary, using standardised methodologies, a range of biomarkers, possibly including metabolic analysis of urine, and quantitative RT-PCR (with appropriate primers to differentiate capsular serotypes, NTHi and *H. haemolyticus)* on nasopharyngeal swabs, serum and blood cultures. Again, this is not entirely straightforward, since neither the presence nor the density of NTHi in nasopharyngeal swabs is correlated with lower respiratory tract NTHi clinical infection (Heidi Smith-Vaughan, Personal Communication, 24 February 2015). Since the majority of cases of CAP are not investigated at all, systematic studies utilising improved case ascertainment, standardised case definitions, diagnostic and sampling techniques, coupled with modern molecular diagnostic techniques could provide an indication of the likely proportion of CAP due to NTHi in any given age group [134].

## 8. Conclusion

NTHi is a well-recognised cause of mucosal infections such as otitis media, sinusitis, and exacerbations of COPD. With the use of sophisticated diagnostic techniques and appropriate molecular identification, it is clear that NTHi are also an important but under-recognised cause of CAP in both children and adults. In countries where Hib has been virtually eliminated by the use of the Hib conjugate vaccine, NTHi are by far the most common cause of *H. influenzae* infections across all age groups, including pneumonia. Improved surveillance of bacteraemic and non-bacteraemic CAP is needed to fully understand the role of NTHi in this important group of infections.
